# Deep response to a combination of mTOR inhibitor temsirolimus and dual immunotherapy of nivolumab/ipilimumab in poorly differentiated thyroid carcinoma with *PTEN* mutation: a case report and literature review

**DOI:** 10.3389/fendo.2024.1304188

**Published:** 2024-01-31

**Authors:** Youjin Oh, Joo Hee Park, Trie Arni Djunadi, Zunairah Shah, Liam Il-Young Chung, Young Kwang Chae

**Affiliations:** ^1^Feinberg School of Medicine, Northwestern University, Chicago, IL, United States; ^2^Department of Medicine, John H. Stroger Jr. Hospital of Cook County, Chicago, IL, United States

**Keywords:** poorly differentiated thyroid carcinomas, mTOR inhibitors, immunotherapy, treatment outcomes, case report

## Abstract

Treating advanced thyroid cancer presents challenges due to its resistance to various treatment modalities, thereby limiting therapeutic options. To our knowledge, this study is the first to report the efficacy of temsirolimus in conjunction with dual immunotherapy of nivolumab/ipilimumab to treat heavily treated advanced PDTC. A 50-year-old female initially presented with a rapidly enlarging mass on her right neck. Subsequent diagnosis revealed poorly differentiated thyroid carcinoma, leading to a total thyroidectomy followed by post-operative radioablation therapy. After four years, an examination for persistent cough revealed a recurrence of the disease within multiple mediastinal nodes. Genetic analysis of blood samples uncovered somatic mutations in the tumor, specifically involving *PTEN* and *TP53*. The disease progressed despite palliative radiation, lenvatinib, and nivolumab/ipilimumab therapy. Consequently, temsirolimus, functioning as an mTOR inhibitor, was introduced as an adjunct to the nivolumab/ipilimumab regimen. This combination approach yielded remarkable clinical improvement and disease control for a duration of approximately six months. Temsirolimus likely suppressed the aberrantly activated PI3K/AKT/mTOR signaling pathway, facilitated by the PTEN genetic alteration, thus engendering an effective treatment response. This synergy between targeted agents and immunotherapy presents a promising therapeutic strategy for advanced PDTC patients with limited treatment alternatives. In previous clinical trials, mTOR inhibitors have demonstrated the ability to maintain stable disease (SD) in 65% to 74% for advanced thyroid cancer patients, including those with PDTC. When combined with other targeted therapies, the observed SD or partial response rates range from 80% to 97%. Many of these trials primarily involved differentiated thyroid carcinoma, with diverse genetic mutations. Thyroid cancer patients with alterations in the PI3K/mTOR/Akt appeared to benefit most from mTOR inhibitors. However, no clear association between the efficacy of mTOR inhibitors and specific histologies or genetic mutations has been established. Future studies are warranted to elucidate these associations.

## Introduction

Poorly differentiated thyroid carcinoma (PDTC), a rare histologic type of malignant thyroid tumor comprising 2 to 13% of all thyroid cancers, features an extremely aggressive disease course (1). The reported survival data varies with a five-year overall survival (OS) of 60-85% to a median survival of 3.7-5 years (2–4). An unfavorable prognosis is associated with the pathological tumor size greater than 4cm, extrathyroidal metastases at diagnosis, refractoriness to radioiodine therapy, and lack of effective treatment strategies (4, 5).

The majority of cases are known to be refractory to radioactive iodine (RAI) therapy, traditional chemotherapy, and radiotherapy (6). Multimodal therapy, including surgery and RAI, can be beneficial in prolonging survival in patients with locoregional disease (7). However, few therapeutic options are available for patients with advanced PDTC who are not eligible for surgical resection or RAI therapy.

Accordingly, immunotherapy or targeted therapy based on up-front genetic information is being examined. The most frequently altered genes in PDTC are *TERT* and *TP53* - others being *BRAF, RAS, AKT, PTEN, and PIK3CA* - and are associated with pathways like *MAPK* and *PI3K-AKT* (8, 9).

We describe the first case of combination therapy with temsirolimus and dual immunotherapy of nivolumab/ipilimumab in PDTC with *PTEN* mutation. This is the first evidence that illustrates targeting *PTEN* with temsirolimus can induce a favorable treatment response among PDTC patients. In particular, temsirolimus added to immunotherapy showed a synergistic effect, eliciting a favorable response, which was not achieved with only immunotherapy. Given the lack of literature on the outcomes and management of PDTC, this case can contribute to managing PDTC patients harboring a specific mutation with targeted therapy.

## Case presentation

The patient is a 50-year-old female, a former smoker, who had noted a rapidly enlarging mass on the right side of the neck. Fine needle aspiration (FNA) revealed poorly differentiated thyroid cancer. She underwent total thyroidectomy, which revealed a 4.0cm long axis and confirmed poorly differentiated thyroid cancer on the right thyroid. Vascular and capsular invasion were present, but resection margins were clear, and no lymph node dissection was performed. Her left thyroid was benign. She received post-surgical RAI treatment with 153 mCi I-131 and was on levothyroxine post-treatment. Serum thyroglobulin (Tg) level was low before RAI and undetectable post-therapy. Surveillance thyroid sonography showed stable bilateral thyroid bed nodules with sizes below 0.5 cm. No recurrence was found on PET-CT at 18 months and chest CT at 30 months after diagnosis.

**Figure 1:**
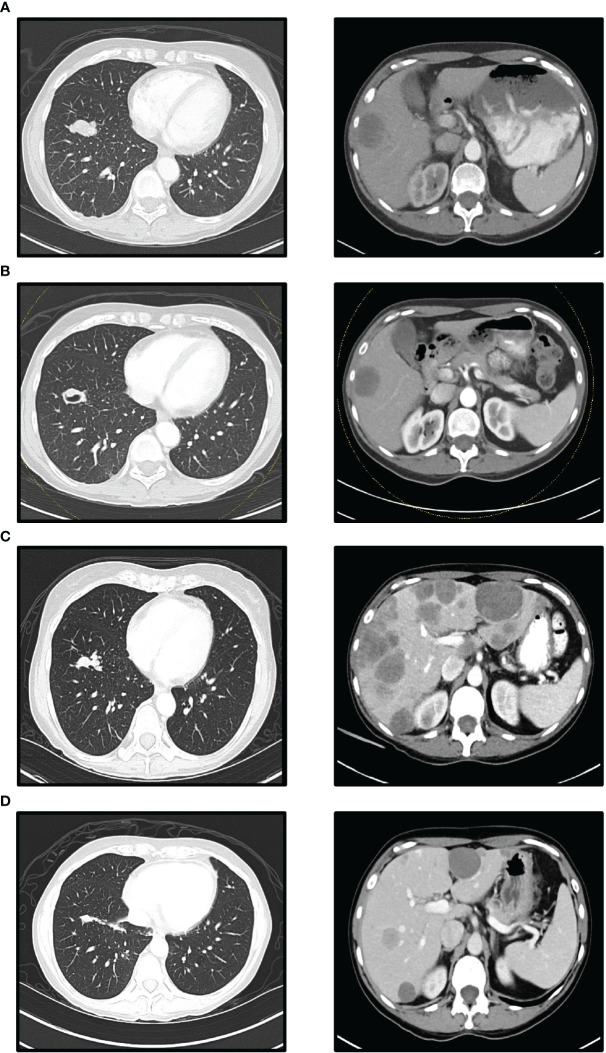
Computed Tomographic (CT) scans (left lower lobe mass and liver metastases): **(A)** Before nivolumab/ipilimumab treatment. **(B)** After 42 days of nivolumab/ipilimumab treatment. **(C)** Before temsirolimus add-on. **(D)** After 90 days temsirolimus add-on **(A, B)** show when nivolumab/ipilimumab treatment resulted in a stable disease response for 7 weeks. **(C)** shows PD response 4 months after starting nivolumab/ipilimumab treatment. The largest liver lesion in **(C)** measured 88 mm in diameter at its longest. **(D)** shows significantly reduced lung and liver metastases 3 months after treatment with temsirolimus and nivolumab/ipilimumab.

Four years after total thyroidectomy, she presented with persistent cough, shortness of breath, and wheezing. Chest CT revealed numerous large lymph nodes, 5.7 cm in the mediastinum, 1.5cm in the left supraclavicular, and multiple 3.0 cm nodules in the right middle and lower lobes. Endobronchial ultrasound bronchoscopy biopsy (EBUS) confirmed poorly differentiated thyroid carcinoma with necrosis, which was clinically suspected as metastasis or recurrence. A positron emission tomography (PET) scan confirmed multiple fluorodeoxyglucose (FDG) avid lesions, suggesting nodal, pulmonary, hepatic, and osseous metastatic disease. The baseline sum of the target lesions was 18.1 cm, with the liver lesion measuring 3.8 cm (**Figure 1A**). Serum Tg was 0.11ng/mL, thyroid-stimulating hormone (TSH) 0.01μIU/mL, and free thyroxine (fT4) 1.2ng/dL.

**Figure 2:**
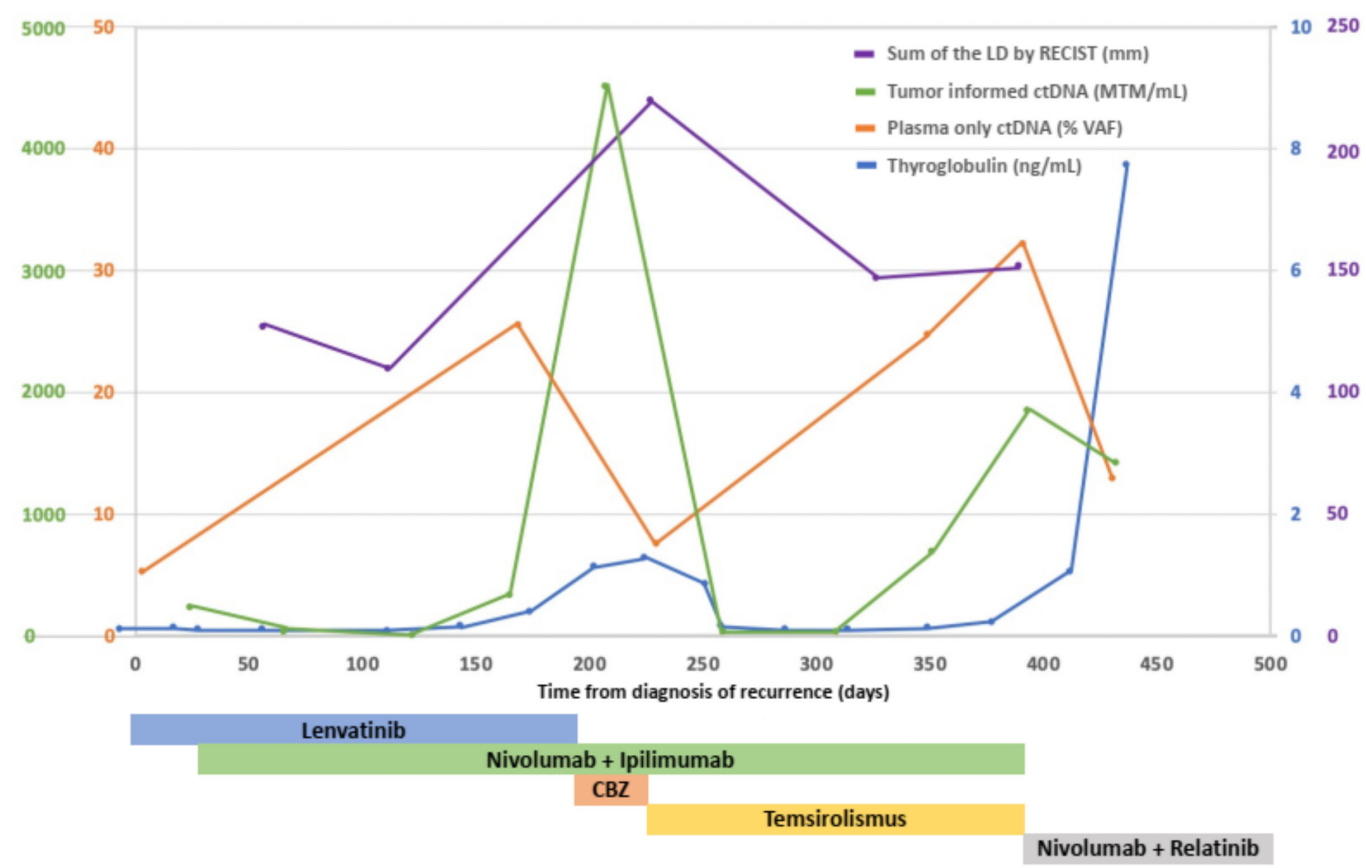
Sum of the LD of target lesions by RECIST (mm), serum thyroglobulin levels (ng/mL), mean ctDNA molecules (MTM) per mL of patient’s plasma measured by tumor-informed circulating tumor DNA (ctDNA), variant allele fraction (VAF) of somatic mutations measured by plasma-only circulating tumor DNA (ctDNA) in the treatment course. After adding temsirolimus to nivolumab and ipilimumab, the patient’s sum of the LD of target lesions, serum thyroglobulin level, and ctDNA measured by tumor informed ctDNA assay decreased remarkably. CBZ, cabozantinib; LD, longest diameters.

Circulating tumor DNA (ctDNA) was analyzed using two different assays (**Figure 2**). The tumor-informed ctDNA assay (assay-t) measures ctDNA molecules detected per mL of the patient’s plasma based on 16 somatic variants selected from whole-genome sequencing of the cancer sample. A 234.21/mL of MTM was detected in our patient at the time of recurrence. The plasma-only ctDNA assay (assay-p) detecting somatic alterations in the circulating cell-free DNA isolated from the patient’s blood specimen demonstrated alterations in *PTEN* L194fs and *TP53* F270S with undetectable microsatellite instability (MSI). She received palliative radiation for impending airway compromise due to metastatic lymphadenopathy and was started on lenvatinib 20 mg daily. Patient had some insomnia, voice changes, and mild fatigue, but tolerated the treatment well. However, the regimen was discontinued due to the progression of the disease after a month.

A new regimen of nivolumab (3 mg/kg every two weeks) and ipilimumab (1 mg/kg every six weeks) was started (10). Follow-up CT scan after two months showed stable disease with a sum of target lesion of 15 cm, and -17.1% change from baseline (RECIST 1.1; **Figures 1A, B**). Lenvatinib was continued with the regimen for a synergistic effect. Lenvatinib was discontinued for two weeks due to toxicities, including mouth sores, hand and foot syndrome, and fatigue.

Four months after combination therapy of lenvatinib and nivolumab/ipilimumab, a hard and painful bump appeared on the right forehead. MRI showed extensive osseous metastatic disease in the thoracolumbar spine and bony pelvis. The patient reported excruciating pain in her back and hip, for which she was admitted for pain control. Assay-t showed elevated MTM to 334.72/mL. Tg levels were elevated to 0.40 ng/mL. Progression and toxicity led to the discontinuation of lenvatinib, and cabozantinib 60 mg was added the following month. Follow-up scans showed progression, with the sum of target lesions at 23.3 cm, a 55.3% increase from the previous CT (**Figure 1C**). The caudate lobe lesion of the liver showed greatest enlargement from 2.1 to 8.8 cm. Tg was elevated to 1.27 ng/mL, and cabozantinib was discontinued due to toxicity.

Thus, temsirolimus at a dosage of 25 mg per week was incorporated into the nivolumab/ipilimumab treatment regimen. The patient reported regaining energy and appetite. Her right forehead nodule disappeared on exam. A follow-up assay-p conducted the following week also showed a 70% decrease in variant allele fraction (VAF), from 25.6% to 7.5% for *TP53* F270S and 19.8% to 6.3% for *PTEN* L194fs. In addition, her Tg had decreased to 0.84 ng/mL, then to 0.16 ng/mL in a month. A follow-up assay-t showed a decrease in MTM from 4526.97/mL to 29.41/mL at three months. Subsequent 1-month follow-up showed stable MTM at 26.00/mL. CT scan also showed partial response with the sum of the target lesion of 15.1cm and a -35.2% change from previous CT (**Figure 1D**). Tg levels remained in the low range.

The patient has demonstrated stable disease throughout treatment, exceeding six months. During the administration of temsirolimus, the patient initially encountered pancytopenia (hemoglobin 7.1, platelet 318k), a side effect that improved after a dose adjustment to 15mg weekly. Additionally, the patient reported intermittent ankle swelling and facial acne, however these side effects were well-tolerated, and treatment was never suspended. In particular, the patient’s clinical recovery enabled her to regain an independent lifestyle while undergoing temsirolimus treatment. This resurgence in well-being facilitated meaningful life experiences, including attending her child’s high school graduation—a testament to the qualitative impact of the treatment.

The patient experienced a relapse after six months of receiving the combination of temsirolimus and nivolumab/ipilimumab. Subsequently, a new course of treatment was initiated, combining nivolumab with relatinib. However, patient did not improve. Her symptoms worsened rapidly, and she was placed in hospice care, where she died about three months after discontinuing temsirolimus.

## Methods

Research and literature were searched through the PubMed database by using the following keywords: thyroid cancer, thyroid neoplasm, mTOR inhibitor, case report, case series, observational study, prospective study, retrospective study, intervention study, and clinical trial. We summarized the case reports, observational studies, and clinical trials where mTOR inhibitors alone or in combination with other treatments were exploited as a treatment for thyroid cancer.

## Discussion

We report the first case of effective treatment with temsirolimus combined with dual immunotherapy of nivolumab/ipilimumab in RAI refractory PDTC with multiple metastases. The effectiveness of mTOR inhibitors among other types of thyroid cancers, including anaplastic thyroid cancers (ATC) and medullary thyroid cancer (MTC) have been reported. However, this is the first report that *PTEN* was targeted with mTOR inhibitors like temsirolimus combined with immunotherapy in PDTC.

The recent advancements of targeted therapies, particularly multi-targeted tyrosine kinase inhibitors (TKIs) have significantly impacted the treatment of metastatic thyroid cancer. Despite this progress, the response to TKIs varies widely among patients with thyroid cancer. For differentiated thyroid cancer (DTC), OS was reported to be 22.2 years in the TKI group compared to 5.6 in the untreated group (11). In the DECISION trial, which led to the FDA approval of sorafenib for treating RAI refractory thyroid cancer, 207 of DTC patients with 11.6% (n = 24) PDTC, showed 12.2% of overall response rate, with further analysis showing poorer PFS in PDTC compared to PTC (NCT00984282) (12). In the SELECT trial, lenvatinib achieved 18.3 months of median PFS compared to 3.6 months for placebo in 261 advanced thyroid cancer patients refractory to I-131including 10.7% (n=28) of PDTC (NCT01321554) (13). The COSMIC 311 trial demonstrated that cabozantinib significantly prolonged PFS among patients with radioiodine-refractory DTC, without PDTC, who lack a standard of care, leading to the FDA approval (NCT03690388) (14). However, for ATC, a phase II trial with lenvatinib was halted due to futility, as the minimum objective response rate threshold of 15% was not met in the interim analysis of 20 patients (15). Moreover, almost all patients eventually showing progression on TKIs (13, 16). The resistance mechanism involves the acquisition of new mutations triggering overactivation of pathways or evoking alternate pathways to bypass the drug’s action (17). Additionally, not all patients with thyroid cancer harbor actionable genetic aberrations. For instance, *BRAF* V600E mutations were found in only 33% of PDTCs, limiting the efficacy of BRAF-targeted therapies (18).

The immune checkpoint inhibitor therapy, either in combination with TKIs or as a single agent, is also actively underway among aggressive thyroid cancers. In a phase II study with nivolumab and ipilimumab, one out of four PDTCs and three out of ten ATCs achieved PR (NCT03246958) (19). The phase II ATLEP trial showed that the combination of lenvatinib/pembrolizumab yielded a median PFS of 20 months in 8 metastasized PDTC patients and 9.5 months among 27 metastasized ATC patients (20). However, in the phase II national cancer institute trial combining cabozantinib with nivolumab and ipilimumab, interim results did not indicate an efficacy advantage over the currently approved cabozantinib monotherapy in RAI refractory DTC with progression on one prior vascular endothelial growth factor receptor targeted therapy. The objective response rate within 6 months was 10% among 11 patients, including 2 PDTC (NCI#10240) (21).

Given the limited evidence regarding the efficacy of targeted therapies and immunotherapy for PDTC and the aggressive nature of the disease, there is a clinical unmet need for new therapeutic options catering to patients with PDTC refractory to conventional therapies.

We systematically searched clinical trials investigating mTOR inhibitors combined with or without other agents or not among advanced thyroid cancer patients. A total of 7 clinical trials, three retrospectives, one case report, and one preliminary report were found through a literature search using PubMed (National Library of Medicine, Bethesda, MD, USA). In total, eight clinical trials and one retrospective study are analyzed and described in **Table 1** (23–31). One case report and one retrospective study were excluded from the table as their patients overlapped with the clinical trials (32, 33). The other retrospective study and preliminary report were excluded from the review as the data on the efficacy of mTOR inhibitor alone or combined with other treatments were not retrievable (34, 35). One clinical trial was excluded as it was reported as an abstract only (36).

**Table 1:** Summarization of clinical trials evaluating the efficacy of mTOR inhibitor in thyroid cancer patients.

Author, year	Study type^a^	Sample size^b^	Disease description	Regimen	Response	Details
Fury et al., 2012 (22)	Phase I	7/30 PTC 4 OCA 1 MTC 2	Advanced solid tumor not curable by surgery or radiation therapy	Everolimus 10mg/day orally (days 1 – 21) and cisplatin 20mg/m^2^ IV (days 1, 8, and 15)	SD 1 (PTC) ^c^	Advanced metastatic PTC patients completed 14 times of a 28-day cycle (everolimus monotherapy after cycle 6) with SD
Lim et al., 2013 (23)	Phase II NCT01164176	40 PTC 16 FTC 8 MTC 9 ATC 6 PDTC 1	Locally advanced or metastatic	Everolimus 10mg/day orally	CR 0 (0%) PR 2 (5%) SD 29 (76%) PD 7 (18%) Not evaluable 2 (5%)	Median duration of therapy 6.7 months (range 0.7 – 21.1) Median PFS 47 weeks (95% CI 14.9 – 78.5) OS has not yet been reached
Jain et al., 2015 (24)	Phase I	6/62	unresectable or metastatic cancer, with no standard therapy available that increased survival by at least 3 months	Temsirolimus 15mg/kg IV (days 1, 8, and 15), topotecan 2.8 mg/m^2^ IV (day 1 and 8), and bortezomib (0.6 mg/m^2^) IV (days 1, 4, 8, and 11)	PR 1 (PTC) ^c^ SD 1 (FTC)	The patient with PTC showed best RECIST response of -49% at 13 months and progressed at 15 months post-treatment. The patient with FTC showed OS and PFS for more than 13.2 months.
Manohar et al., 2015 (25)	Retrospective	15 PTC 9 FTC 2 OCA 4	Advanced metastatic cancer ^d^	Sirolimus (4 or 6 mg daily) and cyclophosphamide (100 or 150 mg Monday through Friday, biweekly) ^e^	PD 11 (73%) ^c^	The one-year OS probability 0.70 (95% CI 0.49 – 1.00) The one-year PFS probability 0.45 (95% CI 0.26 – 0.80)
Schneider et al., 2017 (26)	Phase II	28 PTC 6 FTC 7 FVPTC 3 FTC-OV 8 PDPTC 3 ATC 1	progressive metastatic or locally advanced RAI refractory differentiated thyroid cancer	Everolimus 10mg/day orally	CR 0 (0%) PR 0 (0%) SD 17 (65%) PD 11 (39%)	Median duration of treatment 11 months (range 1-38) Median PFS 9 months (95% CI 8 – 14) Median OS 18 months (95% CI 6 – 29) Median duration of SD 78 months (range 9 – 150)
Sherman et al., 2017 (27)	Phase II	36 PTC 22 FTC 1 OCA 5 PDTC 6 ATC 2	Surgically inoperable and/or recurrent/metastatic RAI refractory thyroid cancer	Temsirolimus 25mg weekly IV and sorafenib 200mg twice daily orally	CR 0 (0%) ^f^ PR 8 (27%) SD 21 (70%) PD 1 (3%)	Median duration of treatment 6.7 months (range 0.5 – 63) Median OS 24.6 months for all patients 33.7 months for patients with PR 24.0 months for patients with SD 1.2 months for patients with PD PFS at one year of 30.5% For six patients who discontinued sorafenib, temsirolimus was continued for a range of 1.1 - 38 months
Hanna et al., 2018 (28)	Phase II	50 PTC 14 FTC 5 OCA 13 PDTC 1 MTC 10 ATC 7	Locoregionally advanced or metastatic, incurable thyroid cancer For DTC, refractory to RAI	Everolimus 10mg/day orally	CR 0 (0%) PR 3 (6%) SD 37 (74%) PD 7 (14%) Unevaluable 3 (6%)	Median OS 32.7 months (95% CI 18.7 – 61.3+) Median PFS 12.5 months (95% CI 7.3 – 17.5)
Harvey et al., 2020 (29)	Phase I NCT01218555	5/44 ^g^	Advanced thyroid cancer	Everolimus 5mg/day and lenalidomide 10mg/day orally ^h^	PR 1 (20%) SD 3 (60%) PD 1 (20%)	Median TTF 17.4 months (95% CI 1.2 – 30) TTF at one year 60.0% (95% CI 12.6 – 88.2) TTF at two years 20.0% (95% CI 0.8% – 58.2%)
Sherman et al., 2021 (30)	Phase II NCT02143726	OCA 33	Refractory to RAI, PD by RECIST over prior 14 months, no prior treatment with sorafenib or everolimus	Everolimus 5mg/day and sorafenib 400mg twice daily orally (SE group) vs. sorafenib 400mg twice daily orally (S group)	CR + PR 4 (24%) in SE group 3 (19%) in S group	Median PFS 24.7 months (95% CI 6.1 – 33.8) in SE group 9.4 (95% CI 5.5 – no upper) in S group

a NCT number was recorded if the number was available.

b We summarized the number of thyroid cancer patients out of all cancer patients included in the study and the number of subtypes of thyroid cancer as well if data was available.

c In these studies, treatment response for thyroid cancer patients was only available for the patients summarized in the table.

d In this study, 53.3% of the population received treatment above second-line therapy and 14 out of 15 (93.0%) of the patients received prior thyroidectomy.

e In this study, a typical drug dose on initiation was described in the table. The median tolerated dose was 50 mg Monday through Friday, every other week for cyclophosphamide, and 4 mg daily for sirolimus.

f In this study, six patients discontinued treatment before the first scan for reasons other than the progression of the disease.

g In this study, there were 44 patients, including 15 with adenoid cystic carcinoma, 9 with renal cell carcinoma, 5 with thyroid cancer, and 15 with other cancers.

h In this study, five dose cohorts were planned, starting at dose level one with everolimus 5 mg and lenalidomide 10 mg in a 28-day cycle up to maximum doses of 10 mg and 25 mg, respectively.

ATC, anaplastic thyroid cancer, FTC, follicular thyroid carcinoma; FTC-OV, follicular-oncocytic thyroid carcinoma; FVPTC, follicular variant PTC; OCA, Oncocytic carcinoma of the thyroid; MTC, medullary thyroid cancer; PDTC, poorly differentiated thyroid cancer; PDPTC, poorly differentiated PTC; TTF, Time to treatment failure; RAI, radioactive iodine; OS, Overall survival; PFS, Progression-free survival; CR, complete response; PR, partial response; SD, stable disease; PD, progression.

mTOR inhibition has been reported as having promising results in thyroid cancer. Three phase II clinical trials have reported a stable disease rate of 65–76% in those treated with everolimus across different histologies of thyroid cancer (23, 26, 28). The median PFS and OS ranged from 9 to 12.5 months and 18 to 32.7 months, respectively (23, 26, 28). Median time to treatment failure (TTF) of everolimus with lenalidomide was 17.1 months among advanced thyroid cancer (29). In OCA, everolimus combined with sorafenib elicited longer PFS than sorafenib monotherapy (30). Unlike everolimus, temsirolimus has been studied in combination with other therapeutic agents. In a phase II trial, all patients with thyroid cancer, except one with ATC, experienced stable disease, or partial response on the combination of temsirolimus and sorafenib (27). In another phase I trial, patients with PTC and FTC remained without progression longer than a year with temsirolimus, topotecan, and bortezomib treatment (24). A recent study using patient-derived xenograft mouse models of oncocytic carcinoma of the thyroid (OCA) with upregulated mTOR signaling showed tumor suppressing effect of mTOR inhibitors for both primary tumor and metastasis (37).

The association between the efficacy of mTOR inhibitors and histology is unclear. In a study, no significant difference was noticed according to histology, but a durable clinical response was noticed in a high proportion of DTC and MTC patients (23). In another study, the median PFS of patients with PDTC/ATC was the lowest of any subgroup in one study (28). None of the patients with ATC showed a response on everolimus in the other study (26). For PDTC, the PFS was reported for two patients being 6-15 months and 46 weeks from two studies, respectively (23, 28). Among six patients with PDTC treated with temsirolimus with sorafenib, four patients showed partial response and two patients demonstrated stable disease with 8 months of median duration on treatment (27). Collectively, patients with ATC seem to show a relatively less favorable response to mTOR inhibitors compared to the other histologies.

Thyroid cancer patients with PI3K/mTOR/Akt mutations, like our case, showed favorable responses to mTOR inhibitors, consistent with others (28). For everolimus, the median PFS of ATC/DTC patients was 15.2 months for those who had PI3K/mTOR/Akt alterations (*TSC2*, *FLCN*, or *NF1*), compared to 2.8 months for all PDTC/ATC patients (28). One PDTC patient with *TSC2* missense mutation achieved up to 15 months of PFS and more than 21 months of OS (28). One patient with follicular thyroid cancer (FTC) harbored *PTEN* (c.404T>A, p.(Ile135Lys)) and *HRAS* (c.182A>G, p.(Gln61Arg)) mutations had PFS of 41 weeks and OS of 113 weeks for everolimus (26). For temsirolimus and sorafenib combination therapy, one patient with *PTEN* mutation showed a PFS of 22.5 months with maximum disease shrinkage of -51% (27).

*TP53* and *PTEN* mutations are reported in 8% and 4% of PDTC, respectively (18, 38). Phosphatase and tensin homology encoding chromosome 10 (PTEN) is a tumor suppressor gene that encodes a submolecule in the signaling pathway of PIK3CA-PTEN-Akt-mTOR. Currently, there is no approved targeted therapy for *PTEN* mutation for any solid tumors, including thyroid cancer. However, temsirolimus inhibiting *mTOR* (a serine-threonine kinase), a downstream effector of PTEN, showed efficacy combined with dual immunotherapy in our patient. In a study involving another mTOR inhibitor, everolimus, the median PFS was 2.8 months in PDTC/ATC patients, but 15.2 months in patients with PI3K/mTOR/Akt-mutation, highlighting that an optimal therapeutic response of mTOR inhibitor correlates with the targetable mutations (28).

However, it should be noted that the impact of other mutations than PI3K/mTOR/Akt pathway on the efficacy of mTOR inhibitors remains unclear. We summarized the genetic alterations and treatment response to everolimus among thyroid cancer patients in **Supplementary Table 1**. In one study, two patients with a *BRAF* c.1799T>A mutation for PTC had an approximately 11-month difference in PFS on everolimus (26). In another study with temsirolimus with sorafenib, 3 out of 10 PTC patients with only *BRAF* mutations showed the longest PFS/OS of more than 21 months, while other seven patients with multiple gene mutations, even those in the PI3K/mTOR/Akt pathway, had a shorter PFS/OS (28). Similarly, two patients with OCA without any mutation showed the longest PFS/OS of more than 21 months compared to temsirolimus and sorafenib (28). This suggests that further research is needed to further evaluate the association of genetic mutation with the effectiveness of mTOR inhibitors to select patients who may benefit from mTOR inhibitors.

Common adverse events associated with mTOR inhibitors were hematologic, metabolic, pulmonary, and cutaneous side effects (39). In one clinical trial, the dosage of everolimus was reduced among 45% of the patients mostly due to fatigue, pneumonitis, and stomatitis (NCT01118065) (40). Dose reduction for temsirolimus was also necessary among patients with renal cell carcinoma to control thrombocytopenia, neutropenia, elevated triglycerides, stomatitis, hyperglycemia, rash, elevated liver aminotransferases, and pneumonia (39). Our patient also suffered pancytopenia from temsirolimus, which resolved after a dose reduction from 25mg weekly to 15mg weekly.

Furthermore, diagnosis based on ctDNA information can directly guides selecting targetable mutations. ctDNA is known to be detected in more than two-thirds of thyroid cancer patients and it has been speculated to be reflective of disease burden, allowing rapid assessment of response to targeted therapies (41). Utilizing two different ctDNA assays, tumor-informed and plasma-only, to monitor the molecular landscape dynamics of the patient’s tumor revealed targeted mutations and showed treatment response. ctDNA measured by MTM (/mL) and VAF (%) of targeted mutation demonstrated a significant drop after initiating temsirolimus, consistent with radiographic responses. Considering that thyroid cancer is known to recur within five years where high-risk features were present, molecular assays may potentially have a role in guiding innovative treatments and benefiting patients (42).

Generalizing the results of our study presents challenges due to its nature as a single case report. Furthermore, determining the precise contribution of each drug to the treatment response is complex, given the combination therapy involved an mTOR inhibitor and dual immunotherapy. Given disease progression after six months in our case, the absence of a further analysis our understanding of mTOR inhibitors remain limited. Further analysis investigating resistance mechanisms along with changes in genomic alterations are warranted. These limitations highlight the need for larger-scale studies encompassing diverse cases and in-depth analyses to discern the nuanced dynamics at play in this therapeutic approach.

## Conclusion

We report the first case of a patient with recurrent metastatic PDTC with *PTEN* mutation who responded well to combination therapy of mTOR inhibitor (temsirolimus) and dual immunotherapy (nivolumab/ipilimumab), who was previously refractory to radiotherapy and other medical therapies, including lenvatinib and cabozantinib. This represents a novel treatment approach for patients with PDTC. Our patient showed a durable response to treatment for over 6 months. This aligns with previous research showing the promising efficacy of mTOR inhibitors among advanced thyroid cancer patients across different histologies and various genetic mutations.

## Data availability statement

The original contributions presented in the study are included in the article/**Supplementary Material**. Further inquiries can be directed to the corresponding author.

## Ethics statement

The studies involving humans were approved by The Institutional Review Board Committee of Northwestern University, Chicago, IL, USA (STU00207117). The studies were conducted in accordance with the local legislation and institutional requirements. The participants provided their written informed consent to participate in this study. Written informed consent was obtained from the individual(s) for the publication of any potentially identifiable images or data included in this article.

## Author contributions

YO: Data curation, Project administration, Writing – original draft, Writing – review & editing, Formal analysis, Investigation, Visualization. JP: Data curation, Validation, Writing – original draft, Writing – review & editing, Investigation. TD: Data curation, Visualization, Writing – original draft, Writing – review & editing, Formal analysis. ZS: Writing – original draft, Writing – review & editing. L-YC: Data curation, Writing – review & editing. YC: Conceptualization, Investigation, Methodology, Resources, Supervision, Writing – review & editing.

## Supplementary material

Supplementary Table 1 Summarization of tumor mutation and treatment response to everolimus among thyroid cancer patients. ^a^ In this study, survival data was expressed with the unit of the week and converted into the month. ATC, anaplastic thyroid cancer; FTC, follicular thyroid carcinoma; FTC-OV, follicular-oncocytic thyroid carcinoma; FVPTC, follicular variant PTC; OCA, Oncocytic carcinoma of the thyroid; MTC; medullary thyroid cancer, PDTC, poorly differentiated thyroid cancer; OS, Overall survival; PFS, Progression-free survival.
