# Supplementary material for: Deep response to a combination of mTOR inhibitor temsirolimus and dual immunotherapy of nivolumab/ipilimumab in poorly differentiated thyroid carcinoma with PTEN mutation: a case report and literature review
Source: Front Endocrinol (Lausanne). 2024 Jan 31;15:1304188. doi: 10.3389/fendo.2024.1304188 (PMC10864638; doi:10.3389/fendo.2024.1304188)
Supplement: Supplementary Table 1 — Summarization of tumor mutation and treatment response to everolimus among thyroid cancer patients. a In this study, survival data was expressed with the unit of the week and converted into the month. ATC, anaplastic thyroid cancer; FTC, follicular thyroid carcinoma; FTC-OV, follicular-oncocytic thyroid carcinoma; FVPTC, follicular variant PTC; OCA, Oncocytic carcinoma of the thyroid; MTC; medullary thyroid cancer, PDTC, poorly differentiated thyroid cancer; OS, Overall survival; PFS, Progression-free survival. [file Table_1.docx]

Supplementary Table 1. Summarization of tumor mutation and treatment response to everolimus among thyroid cancer patients

| **Study** | **Histology** | **Mutation** | **PFS/OS (months)** |
| --- | --- | --- | --- |
| Lim et al., 2016 (32) | ATC | *TSC1* W103X | 3/3^+^ |
| Lorch et al., 2013 (35) | ATC | *TSC2* Q1178* | 18^+^/18^+^ |
| Schneider et al., 2017^a^ (23) | PTC | *BRAF* c.1799T>A, p(Val600Glu) | 33/33 |
|  |  | *BRAF* c.1799T>A, p.(Val600Glu), *AKT1* c.49G>A, p.(Glu17Lys) | 26/26 |
|  |  | *BRAF* c.1796T>A, p(Val600Glu) | 22/36 |
|  |  | *BRAF* c.1799-1801het_delTGA, p(Val600Glu) | 18/22 |
|  |  | *TSC1* c.C163T, p.(Gln55X) | 1/2 |
|  | FTC | *NRAS* c.182A>G, p.(Gln61Arg) | 29/29 |
|  |  | *HRAS* c.182A>G, p.(Gln61Arg), *PTEN* c.404T>A, p.(Ile135Lys) | 9/26 |
|  |  | *NRAS* c.182A>G, p.(Gln61Arg) | 6/23 |
|  |  | *CDKN1A* c.45_57del, p.15-19del | 1/1^a^ |
|  | FTC-OV | *CDKN2A* c.G250T, p.(Asp84Tyr) | 3/5 |
|  | FVPTC | *NRAS* c.183A>T, p.(Glu61His) | 29/29 |
|  |  | *NRAS* c.182A>G, p.(Glu61Arg) | 15/28 |
| Hanna et al., 2018 (24) | PDTC | High copy gain of *EGFR, TP53*  Missense mutation of *KIT, TSC2, TP53, NOTCH2, ATM* | 6-15/21^+^ |
|  | PTC | Missense mutation of *BRAF* | 21^+^/21^+^ |
|  |  | Missense mutation of *BRAF* | 21^+^/21^+^ |
|  |  | Missense mutation of *BRAF* | 21^+^/21^+^ |
|  |  | Missense mutation of *KIT, RET, NOTCH1, ATM*  High copy gain of *EGFR, RB1* | 16-20/21^+^ |
|  |  | Missense mutation of *KRAS, TSC2, NOTCH1, RB1* | 6-15/21^+^ |
|  |  | Missense mutation of *MET, PIK3CA, NOTCH2, ATM*  High copy gain of *EGFR* | 6-15/21^+^ |
|  |  | Missense mutation of *BRAF, RET, TSC2, TP53, NOTCH1, NOTCH2* | 6-15/16-20 |
|  |  | Missense mutation of *BRAF, PIK3CA, mTOR, TSC2, TP53, ATM* | 6-15/6-15 |
|  |  | Missense mutation of *TSC2, NF1, NOTCH2, ATM* | 6-15/6-15 |
|  |  | Missense mutation of *KIT, KRAS, NF1, TP53, NOTCH2* | < 6/6-15 |
|  | OCA | Missense mutation of *ATM, CREBBP, ARID2* | 21^+^/21^+^ |
|  |  | None | 21^+^/21^+^ |
|  |  | None | 21^+^/21^+^ |
|  |  | Missense mutation of *TSC2* | 16-20/21^+^ |
|  |  | Missense mutation of *NOTCH2, ATM* | 16-20/21^+^ |
|  |  | Missense mutation of *NRAS, RET, CDK4* | 6-15/16-20 |
|  |  | Missense mutation of *TSC2, TP53, NOTCH2, FLT3*  Indel + missense mutation of *FLCN* | 6-15/6-15 |
|  |  | Missense mutation of *TSC2, TP53, NOTCH2, FLT3*  Indel + missense mutation of *FLCN* | 6-15/6-15 |
|  |  | Missense mutation of *EGFR, TP53, NOTCH2*  High copy gain of *EGFR* | 6-15/6-15 |
|  |  | Missense mutation of *ERBB4, NOTCH2, CREBBP* | < 6/< 6 |
|  |  | Missense mutation of *BRAF, TP53*  Indel + missense mutation of *NOTCH2* | < 6/< 6 |
|  | FTC | Missense mutation of *PIK3CA, TP53, NOTCH2* | 6-15/21^+^ |
|  |  | Indel+missense mutation of *EGFR, CARD11*  Missense mutation of *CREBBP, MYC, ATM, NOTCH2, RET, MET* | 6-15/21^+^ |
|  |  | Missense mutation of *EGFR, KIT, NOTCH2, ATM*  High copy gain of *BRAF, EGFR* | 6-15/6-15 |
|  | ATC | Missense mutation of *RET, NF1, TP53, NOTCH2, ATM* | 21^+^/21^+^ |
|  |  | Truncating mutation of *TSC2*  Missense mutation of *TP53, MYC*  Indel + missense mutation of *NOTCH2* | 16-20/21^+^ |
|  |  | Missense mutation of *TP63, NOTCH2, MLL2, ARID2, EP300*  High copy gain of *EGFR* | < 6/6-15 |
|  |  | Truncating mutation of *FLCN*  Missense mutation of *MET, NRAS, TP53, NOTCH2, ATM, CREBBP* | < 6/< 6 |
|  |  | Missense mutation of *KIT, MYC*  Indel + missense mutation of *NOTCH2* | < 6/< 6 |
|  |  | Missense mutation of *BRAF, KIT, ATM, MLL2* | < 6/< 6 |
|  | MTC | Missense mutation of *RET* | 21^+^/21^+^ |
|  |  | Missense mutation of *RET, TP53*  Truncating mutation of *NF1* | 21^+^/21^+^ |
|  |  | Missense mutation of *MET, RET, NOTCH4*  High copy gain of *EGFR, RET* | 21^+^/21^+^ |
|  |  | Missense mutation of *RET, TP53, NOTCH2*  Truncating mutation of *TP63* | 6-15/16-20 |
|  |  | Missense mutation of *RET, NOTCH2, ATM, FLT3*  High copy gain of *CCND1* | 6-15/6-15 |
|  |  | Missense mutation of *RET, TSC2, ATM* | 6-15/6-15 |
|  |  | Missense mutation of *mTOR*  High copy gain of *EGFR* | 6-15/6-15 |
|  |  | Missense mutation of *RET, NOTCH1, NOTCH2, SMARCA4*  High copy gain of *EGFR* | < 6/21^+^ |

^a^ In this study, survival data was expressed with the unit of the week and converted into the month.

ATC, anaplastic thyroid cancer; FTC, follicular thyroid carcinoma; FTC-OV, follicular-oncocytic thyroid carcinoma; FVPTC, follicular variant PTC; OCA, Oncocytic carcinoma of the thyroid; MTC; medullary thyroid cancer, PDTC, poorly differentiated thyroid cancer; OS, Overall survival; PFS, Progression-free survival;
